# Principles and Practices of Neurodevelopmental Assessment in Children: Lessons Learned from the Centers for Children’s Environmental Health and Disease Prevention Research

**DOI:** 10.1289/ehp.7672

**Published:** 2005-06-24

**Authors:** Kim N. Dietrich, Brenda Eskenazi, Susan Schantz, Kimberly Yolton, Virginia A. Rauh, Caroline B. Johnson, Abbey Alkon, Richard L. Canfield, Isaac N. Pessah, Robert F. Berman

**Affiliations:** 1 University of Cincinnati College of Medicine, Division of Epidemiology and Biostatistics, Department of Environmental Health, and the Cincinnati Children’s Environmental Health Center, Cincinnati, Ohio, USA; 2 Center for Children’s Environmental Health Research, School of Public Health, University of California at Berkeley, California, USA; 3 The Friends Children’s Environmental Health Center, University of Illinois, Urbana-Champaign, Illinois, USA; 4 Children’s Environmental Health Center, Cincinnati Children’s Hospital Medical Center and University of Cincinnati College of Medicine, Cincinnati, Ohio, USA; 5 Columbia Center for Children’s Environmental Health, Columbia University, New York, New York, USA; 6 Pediatric Neuropsychology Group, Berkeley, California, USA; 7 Department of Family Health Care Nursing, School of Nursing, University of California, San Francisco, California, USA; 8 Division of Nutritional Sciences, College of Human Ecology, Cornell University, Ithaca, New York, USA; 9 Department of Molecular Biosciences, and; 10 Department of Neurological Surgery, University of California at Davis, Davis, California, USA

**Keywords:** behavior, development, National Children’s Study, neurotoxicology, study design

## Abstract

Principles and practices of pediatric neurotoxicology are reviewed here with the purpose of guiding the design and execution of the planned National Children’s Study. The developing human central nervous system is the target organ most vulnerable to environmental chemicals. An investigation of the effects of environmental exposures on child development is a complex endeavor that requires consideration of numerous critical factors pertinent to a study’s concept, design, and execution. These include the timing of neurodevelopmental assessment, matters of biologic plausibility, site, child and population factors, data quality assurance and control, the selection of appropriate domains and measures of neurobehavior, and data safety and monitoring. Here we summarize instruments for the assessment of the neonate, infant, and child that are being employed in the Centers for Children’s Environmental Health and Disease Prevention Research, sponsored by the National Institute of Environmental Health Sciences and the U.S. Environmental Protection Agency, discuss neural and neurobiologic measures of development, and consider the promises of gene–environment studies. The vulnerability of the human central nervous system to environmental chemicals has been well established, but the contribution these exposures may make to problems such as attention deficit disorder, conduct problems, pervasive developmental disorder, or autism spectrum disorder remain uncertain. Large-scale studies such as the National Children’s Study may provide some important clues. The human neurodevelopmental phenotype will be most clearly represented in models that include environmental chemical exposures, the social milieu, and complex human genetic characteristics that we are just beginning to understand.

The purpose of this article is to present lessons learned in neurodevelopmental assessment of children from the Centers for Children’s Environmental Health and Disease Prevention Research (Children’s Centers) and from previous research. These comments and recommendations are products of the authors’ experiences within their centers and their past works in the area of environmental neurotoxicology and teratology. We present both missteps and achievements in the hope that our collective experience can help guide in the planning and implementation of the National Children’s Study.

## The Central Nervous System as a Critical Organ

A fundamental lesson learned from studies of the effects of environmental exposures to the fetus and child is that the developing brain is one of the organs in the human body most sensitive to damage. Functional manifestations ranging from frank mental retardation to milder learning disabilities are the most common class of birth defects (Lipkin 1991), although in most cases the specific etiology is unknown (Kallen 1988). Researchers have speculated that some cases of uncertain etiology may be due to environmental chemical exposures (Rees et al. 1990). The maturation of the central nervous system requires a more complex sequence of processes than any other structure, making this organ uniquely vulnerable to environmental influences (Rodier 1994, 2004).

National and international agencies have focused on the risks of early exposure to several major environmental contaminants such as lead, methylmercury, and polychlorinated biphenyls (PCBs) on neurodevelopment [Agency for Toxic Substances and Disease Registry (ATSDR) 2000; International Programme on Chemical Safety (IPCS) 1995; National Research Council 2000]. Although there is a paucity of data on the risks of lower-level pesticide exposures on human neurodevelopment, there are substantial animal model data and limited human developmental data that these poisons may affect intrauterine growth and have functional teratogenic properties as well (Guillette et al. 1998; Weiss 1997; Weiss et al. 2004).

## Initial Considerations

### Timing of neurodevelopmental assessment.

The vast and rapid growth of the child’s neurobehavioral repertoire from birth through adolescence means that functional expressions of earlier-damaged systems may not be present or accessible at any given moment in time. For example, finding no effects on infant behavior cannot be regarded as conclusive evidence that the toxic agent has had no impact. Negative findings in the preschool period are also inconclusive. A toxicant may damage higher cortical centers that are associated with neurocognitive processes that are not yet functional or only marginally functional in a preschooler. In older children, a wider and more differentiated range of abilities can be examined, scores on psychometric measures are more precise and reliable, and early academic performance and social functioning outside of the home environment can be evaluated. This provides the rationale for long-term prospective longitudinal studies of cohorts recruited prenatally or at birth as the optimal design to ascertain neurobehavioral deficits in relation to exposure to environmental chemicals (Krasnegor et al. 1994). The literature on lead provides a model example of early exposure associated with higher-order neuropsychological and behavioral dysfunctions in older children and adolescents (e.g., Bellinger et al. 1992; Burns et al. 1999; Needleman et al. 1990; Tong et al. 1996). Long-term neurodevelopmental consequences have also been observed in a cohort study of children exposed *in utero* to PCBs (Jacobson and Jacobson 1996, 2003). Evaluations at earlier ages can and have revealed changes in cognitive function, but these may be less robust and definitive (Dietrich and Bellinger 1994).

### Biologic plausibility.

A number of factors determine the transplacental passage of environmental contaminants (Wilson 1977). Toxicants with low molecular weight, lipid affinity, nonpolarity, and low protein binding properties cross the placenta with ease (Slikker and Miller 1994). Unfortunately, most compounds possess one or more of these properties, cross the placenta, and enter the fetal circulation (Beckman and Brent 1999).

The immature blood–brain barrier of the fetus and young infant is more permeable to xenobiotics, and the fetus lacks drug-metabolizing detoxification capacities that are present postnatally (Rozman and Klaassen 1996). The chemical properties of certain toxicants also determine access to brain tissues. Lead imitates calcium ions and therefore crosses the blood–brain barrier with relative ease (Kerper and Hinkle 1997). In the blood stream, methylmercury combines with cysteine, forming a compound that is structurally similar to the essential amino acid methionine. This methylmercury–cysteine compound is actively transported into the endothelial cells in the blood–brain barrier on the methionine carrier and ultimately into the brain on a glutathione carrier (Kerper et al. 1992). The chlorobiphenyls and several classes of pesticides also gain access to brain tissues by virtue of their lipid solubility.

Toxicants linked with growth retardation and maturational delays *in utero* should be considered prime candidates for functional developmental toxicity. Any compound that retards intrauterine somatic growth should be examined as a potential neurobehavioral teratogen (Wilson 1977). Low-level intrauterine exposure to lead, PCBs, and organophosphate pesticides has been associated with lower birth weight, gestational maturity, or reduced head circumference in some prospective studies (e.g., Berkowitz et al. 2004; Dietrich et al. 1987; Eskenazi et al. 2004; Fein et al. 1984; McMichael et al. 1986; Whyatt et al. 2004).

Because of their potential disruption of central nervous system morphoregulation, hormonally active agents should also be considered as candidate neurodevelopmental toxicants. Hydroxylated metabolites of PCBs and related compounds may bind to human transthyretin, the only thyroid-hormone–binding protein synthesized in the brain. By binding to transthyretin, some hydroxylated PCBs may alter brain free thyroxine (T_4_) levels and interfere with central nervous system development and function (Cheek et al. 1999; Seegal 2000). Recent evidence also suggests that PCBs up-regulate several thyroid-hormone–responsive genes that are expressed during periods of brain development (Gauger et al. 2004).

Environmental chemicals with excitatory effects on neurons (prolonged depolarization) are potential developmental neurotoxicants, as well. Examples include nicotine, the organophosphate and carbamate pesticides (Slotkin 1999), and some metals, including lead (Bressler et al. 1999).

### Population factors.

Population factors encompass a host of variables that affect a study’s design, plan of execution, and sensitivity to the potentially adverse neurodevelopmental consequences of exposure to environmental toxicants. Several sometimes highly intercorrelated co-factors are involved, such as socioeconomic status, ethnicity, nutrition, access to medical care and educational resources, language spoken in the home, and cultural milieu. These variables will need to be considered when devising recruitment and long-term retention strategies, and especially when selecting the most appropriate methods of neurodevelopmental assessment. The field of neurobehavioral testing has been at the center of this dilemma as psychologists have struggled with the problem of how to design tests that do not measure only culturally specific information that is quite familiar to children from certain groups or social backgrounds but less familiar to those from different backgrounds. Investigators can use tests that have been adapted for non-English-speaking children and their families. In the United States, this typically involves Spanish translations of extant instruments (e.g., intelligence or speech, language, and memory tests), but the inventory of instruments in Spanish is still quite low. Furthermore, different dialects within the U.S. Spanish-speaking population exist, thus adding another complication. For example, the Children’s Centers at the University of California at Berkeley, Columbia University, and Mount Sinai School of Medicine had Spanish-speaking populations from Puerto Rico, Mexico, and Dominican Republic with different regional dialects. In these cases, it is important to ask the caregiver what language is spoken in the home. All assessments of non-English–speaking children should be done by examiners who are bilingual, preferably with the language of concern (e.g., Spanish) as their native tongue. Piloting of previously translated tests in the population of interest is always essential to determine their suitability. Some nonverbal tests have been considered to be culturally neutral, such as the Raven’s Progressive Matrices (Raven 1998). However, even nonverbal tests may not be completely culturally neutral if the skills needed to complete the task are outside of the cultural experience of the population being evaluated.

One solution to this problem may lie in the use of so-called culture-neutral electrophysiologic, operant learning, or classical conditioning protocols. These types of tests also have the advantage that parallel animal tests that assess the same or similar functional domains are available. The drawback of using these “culture-fair” procedures is that the functional significance of any finding is sometimes difficult to calculate.

The degree of confounding can also vary greatly from one population to another and significantly affect a study’s ability to detect associations between toxicant exposures and various parameters of neurodevelopment. In some populations, the degree of confounding may be so great that, after statistical adjustment, the exposure variable(s) no longer accounts for any further unique (independent) variance in the neurobehavioral data. Lead is a model example where exposure is typically correlated with other suboptimal environmental and sociohereditary factors (e.g., Dietrich et al. 1991). In such cases, any additional attributable developmental risk, beyond that accounted for by confounders, is obscured. This raises the specter of type II error (Needleman and Bellinger 1986).

The problem of confounding is not limited to correlations between a chemical exposure and nonchemical covariables such as socioeconomic status, quality of child rearing, and parental intelligence, among others. In some cases, populations can be exposed to a mixture of compounds that are also intercorrelated. This presents a conceptual and biostatistical challenge when attempting to estimate the independent or combined additive or synergistic effects of multiple chemical exposures (Jacobson 2001). For example, critical reviews of the Faroe Islands study of methylmercury and child development have suggested that the effects attributed to intrauterine exposure to methylmercury might be the consequence of co-exposures to high levels of PCBs [Myers and Davidson 2000; Toxicology Excellence for Risk Assessment (TERA) 1999]. Most other investigations of neurotoxicants in humans have not measured a multitude of exposures but have examined the behavioral toxicity of single toxicants. With the advent of more sensitive biomarkers of environmental exposures, researchers such as those involved in the Children’s Centers and in the National Children’s Study will be facing a new challenge of developing both statistical and toxicologic methods for addressing the impact of multiple exposures. The importance of this has been shown in a recent study in animals demonstrating that the effect of the organophosphate chlorpyrifos was exacerbated by the pharmaceutical agent terbutaline commonly used to prevent preterm delivery (Rhodes et al. 2004).

The problem of confounding in environmental neuroepidemiology has led some to speculate about the advantages of studying chemical or drug exposures in lower risk populations for the purpose of reducing confounding and thus strengthening associations between measures of dose and disease (e.g., Bellinger 1995). However, restricting neurobehavioral studies of environmental toxicants to lower risk populations is not without scientific and ethical drawbacks. U.S. citizens at the lowest levels of social strata tend to suffer the greatest toxic burden. Furthermore, the opportunity to assess the interactions of toxicant exposures with other risk factors may be missed. The effects of prenatal and early postnatal exposure to developmental neurotoxicants may be more severe among the disadvantaged, where nutritional deficiencies, lack of adequate prenatal care, and suboptimal psychosocial environmental factors are likely to be more common.

### Site factors.

Examiners are often compelled to conduct assessments under less than ideal conditions. Inadequate space, lighting, excess ambient noise, and other distractions can invalidate even the most carefully administered protocol. The developmental assessment of an infant or child should be regarded as a controlled experiment, just as in animal models involving behavioral measures.

For neonates and infants, it is especially critical that the clinic environment is conducive to a valid test. For the newborn, lighting, temperature, noise, and any other factors that affect the neonate’s state can determine the validity of the test. For an infant or child of any age, a comfortable, quiet, and well-lighted testing environment is a minimal requirement. Furniture, including chairs and table, should be appropriate for the test and developmental stage of the child. Test materials should be out of the child’s sight but still easily accessible to the examiner to avoid fumbling and other miscues. Infants and some preschool children will often need the security and support of a care-giver during the examination, so accommodations should be made to have another adult in the room, seated in an area where the subject will not be distracted.

In some studies the geographic dispersion of the population may make home or school place testing the only practical option. Indeed, testing sometimes must take place in multiple sites to accommodate families and prevent loss to follow-up. For example, studies in large rural communities (e.g., the Berkeley study of children in the Salinas Valley community) find it necessary to use a recreational vehicle in addition to the clinic site to reach families without any means of transportation and to avoid distractions within the home.

### Child factors.

It would seem obvious that the infant or child should be in an appropriate state or physical condition for assessment. However, this factor is not always given the consideration it deserves. The child should be reasonably well when evaluated with no current infection that is likely to significantly affect performance such as a severe upper respiratory infection or acute otitis media. Any medications the child may be on should also be recorded. It is important to clarify this with the parent before the assessment begins. A child who is too ill to respond appropriately to the demands of the examination should be rescheduled.

Given that many neurodevelopmental testing procedures require normal sensory function, a vision and hearing screen should also precede the examination. In some studies, sensory functions may be core outcome variables. In this case, a more detailed assessment of vision (farsightedness, depth perception, color perception, acuity/contrast sensitivity) and hearing (tympanometry, pure-tone audiometry, central auditory processing) will be indicated. Assessment of audition can be conducted as early as 6 months using methods such as visual reinforcement play audiometry. The auditory brainstem response (ABR) can also be employed to noninvasively measure the electrophysiologic responses of the brainstem auditory pathways (Roizen 1996). The automated ABR is already a routine procedure for screening neonates for hearing problems in a large number of hospitals in the United States, although a program of universal screening has not been implemented [American Academy of Pediatrics (AAP) 1995]. Methods for assessing refractive errors and amblyopia in infants and young children with limited verbal abilities such as the Allen cards and Snellen chart can also be used. However, in the toddler and pre-schooler, observing the child’s response to visually engaging stimuli of various sizes can be just as valuable as a screening tool (Hoon 1996).

These neurosensory data can be used as core outcomes (i.e., if the toxicant in question is expected to affect vision or hearing), as effect modifiers (i.e., a toxicant’s effects on tasks involving visual or auditory processing may be more pronounced among youngsters with preexisting deficits in these areas), or as potential confounders. However, extreme care must be taken when using such data as confounding or control variables. If deficits in neurosensory function are an expression of exposure to the toxicant(s) in question, the resulting overcontrol may lead to a false negative finding or type II error.

Some way of rating the child’s behavior and affect during the test session is also desirable. The child’s response to examiner and test situation, attitude toward self and test performance, work habits and problem-solving style, motor functioning, visual and auditory acuity, oral communication, and mood are some of the factors that could potentially affect performance and should be noted. These data can be entertained as co-factors in outcome analyses, but this may lead to overcontrol. Indeed, behavioral disturbances that are rated by the examiner as limiting the validity of the examination can also be considered as legitimate behavioral sequelae of exposure.

Another factor that can affect a child’s performance is the presence of a parent. A parent provides physical and emotional support for an infant or toddler but can also be a source of disruptive influences on the child’s responses. Although the presence of a familiar caregiver is necessary and desirable for assessment of infants < 2 years of age, the parent or other caretaker should be instructed not to interfere with the presentation of stimulus materials, examiner instructions, or the child’s behaviors during the test. By the later preschool years, the parent should be encouraged to not be present during the examination. If a child absolutely needs the security of a familiar care-giver, the examiner should seat the adult companion outside of the child’s field of vision.

### Quality assurance and quality control.

Analytical laboratories go to great lengths to assure the validity and reliability of their assessments of toxicants in environmental samples and biologic tissues with quality assurance and quality control protocols. Similar issues are germane to neurodevelopmental assessment. Results of individually administered behavioral assessments depend to a great degree on the interaction between the child and examiner. Therefore, it is imperative that the examiner’s contribution be equivalent for all children so that interchild differences in performance can be attributed to child characteristics rather than to some combination of child and examiner characteristics.

Although not always easy to achieve, the following recommendations apply to any longitudinal prospective study of the effects of neurodevelopmental toxicants. Ideally a single examiner should be used at each site or center. The examiner should be well seasoned and familiar with the population of interest. It is not necessary for a doctoral-level psychologist to administer most tests that are likely to be used in these studies (Brandt and van Gorp 1999). Individuals with baccalaureate or master degrees in psychology or related fields with additional training in the standardized administration and scoring of neuropsychological tests can examine children enrolled in a study, under professional supervision [American Academy of Clinical Neuropsychology (AACN) 1999]. The examiner should be blinded to the group membership or exposure status of the child. Children from high-, medium-, and low-level exposure groups (if known) should be tested in a randomly intermixed order. If more than one examiner is used, their comparability in training and technique should be explicitly checked and regularly monitored. In multicenter studies, examiner training should be standardized across sites and regular meetings, and conference calls should be arranged to discuss issues of administration and scoring as they arise.

Dietrich and Bellinger (1994) cite an example of the difficulties that can arise when the second and last guidelines are neglected. In a study of lead-exposed children, the average IQ scores assessed by two relatively inexperienced examiners differed by more than one standard deviation or 15 points (Gregory et al. 1976).

In long-term prospective studies, it is not always possible to rely on a single experienced examiner to test all children. Attrition of staff and requirements for backup psychometricians in the event of leave usually require that a research center employ the services of two or more examiners. Interexaminer differences can be minimized by having the same developmental neuropsychologist train them and by videotaping practice administration sessions to provide feedback to the trainee and assess the presence of any differences in adherence to standardized administration or style that may result in interexaminer variability and measurement error. Videotaping can also provide the opportunity to assess interexaminer reliability in scoring the protocols (Chandlee et al. 2002). In studies spanning several years, monitoring of interexaminer reliability and proficiency should be practiced at regular intervals. This is particularly critical for multicenter studies employing identical neurodevelopmental assessment protocols. Whether one or several examiners are active at a given site, it is imperative that their reliability and proficiency be monitored over the entire course of the study to prevent any drift away from standardized procedures for administration and scoring.

All studies should carefully document quality assurance and control procedures and report intertester and interscorer reliability coefficients where appropriate. As Bellinger (2002) has noted, “the expectations for reporting the reliability of these [neurobehavioral] measurements should be no different from the expectations for reporting exposure (i.e., bio-marker) measurements such as hair-mercury or blood-lead levels.”

Nevertheless, although the Children’s Centers instituted quality control protocols, particularly for the neurodevelopmental assessments, including direct observations or videotaping, insufficient time and resources made it difficult to fully meet the goals of these quality control programs. In our experience, the effort and cost associated with this are frequently underestimated. The National Children’s Study needs to make every effort not to fall short in this critical area.

### Sensitivity and specificity of neurodevelopmental measures.

The terms “sensitivity” and “specificity” have two different meanings in the context of this article. In the evaluation of diagnostic tests, the sensitivity of a measure is defined as the proportion with the abnormality that the test classifies as abnormal (i.e., the proportion of true positives), whereas the “specificity” is the proportion of normal that the test classifies as normal (i.e., the proportion of true negatives). In the selection of neurodevelopmental measures for environmental studies, it is clearly advantageous to include tests that have the best possible prognostic value. This is particularly critical if neurobehavioral end points are incorporated into risk assessments conducted by regulatory bodies (Bellinger 2002). However, in this context we are also concerned with the selection of instruments that tap into neurodevelopmental domains that have been shown to be sensitive to (affected by) exposure to particular environmental toxicants. Of course, this assumes that some information already exists as to the effects of a given compound on the developing nervous system. Sometimes there are no prior human studies available to help dictate the domains that should be examined, and animal studies may not include domains that are unique to human cognition (e.g., language). In cases where there are multiple potential exposures with different mechanisms or loci of effect or when the exact exposures are not known or hypothesized, it may be necessary to use a broad spectrum of assessment tools. However, to the extent such information is available, selection of tests can be based on hypothesis-based inference. Under these circumstances, the focus shifts from “selecting tests” to the nomination of neurobehavioral domains to tap and subsequently to the employment of the best available measures (Bernstein 1994).

A wide range of neurobehavioral assessments has proven to be sensitive to lower level prenatal and early postnatal exposures to environmental contaminants, for example, lead, methylmercury, PCBs, environmental tobacco smoke, and other chemicals. However, the identification of behavioral phenotypes for specific agents has been an elusive goal. Some attempts have been made to differentiate the effects of alcohol exposure *in utero* from deficits in neurobehavior associated with PCBs and other toxicants [e.g., Jacobson 1998; National Institute on Drug Abuse (NIDA) 2000]. However, environmental studies have failed to clearly identify a “behavioral signature” for particular compounds. The best evidence for specific effects probably comes from the literature on PCBs, where deficits in cognitive processing involving visual discrimination and memory appear to be a somewhat consistent finding in exposed infants and preschool children (Darvill et al. 2000; Jacobson et al. 1992). However, toxicants such as lead, methylmercury, and even the chlorobiphenyls seem to affect a wide range of neurobehavioral outcomes without illustrating a convincing degree of consistency across domains from one study to another. The sample’s socioeconomic status; level, pattern, and timing of exposures; nutritional intake; general health; educational opportunities; and the particular instruments that were employed to examine neurodevelopment probably play an important role in between-study differences (Bellinger 1995; Schantz 1996). This could explain why the wide net provided by global and multiple-domain assessments of cognitive development such as IQ have proven to be so consistently sensitive across studies. Because these tests combine subscales that are representative of a broad number of underlying cognitive functions, they are likely to pick up exposure-related deficits across cohorts that differ in their functional expressions of toxicity. However, despite the sensitivity of these tests, their use in environmental studies has been sharply criticized by some reviewers. Critics of these apical measures note that they cannot readily yield information about the affected neural substrates or the complex executive and regulatory processes involved in learning, problem solving, and behavior (Bernstein 1994; Krasnegor et al. 1994; White et al. 1994). Furthermore, although global measures of intellectual functioning have proven to be sensitive measures of exposure to a number of environmental chemicals, this has not been the case for prenatal exposure to alcohol or drugs (NIDA 2000).

### Data safety and monitoring.

There is an ethical responsibility that referral protocols be in place to deal with the needs of children who perform poorly in the course of their participation in the study. Criteria need to be established for referral before data collection begins. Because of the sensitivity of certain tests, only performance on some may result in referral. Also, because of the predictive validity of these tests, a more rigorous criterion may be used at older ages (e.g., scores three standard deviations below the mean up to 24 months and 2 standard deviations afterward). Referrals normally take place through the primary care provider with parental permission. All referral related contacts should be carefully documented and confidentiality closely guarded.

## Neurodevelopmental Assessment in Environmental Studies: Initial Challenges

Constructing a battery of tests for an environmental study of developmental neurotoxicants can be a daunting task. First, the investigator is faced with choosing from a large number of potential end points that can be measured in a prospective study of any given agent or mixture of compounds to which the fetus or child may be exposed. Domains of interest in studies of this kind include overall neurologic status, sensorimotor skills, attention, memory, problem solving (executive functions, organization, and planning), visual–spatial and perceptual skills, speech and language abilities, behavioral problems, and adaptive skills as well as more global indices of intellectual attainment and academic achievement. Ideally, as stated above, the choice of neurobehavioral domains and the tests used to index them should be determined by what is known about the impact of a particular environmental agent on the development of specific cognitive, neuromotor, and behavioral features. However, as previously discussed, this is not always easily accomplished because of the lack of evidence for specific behavioral phenotypes. In this regard, animal studies have potential for identifying key functional domains that are affected by exposure and would be relevant to assess in human populations. For example, the Children’s Center at the University of Illinois is using studies in animals exposed to PCBs and methylmercury during gestation to identify specific aspects of cognitive, sensory, and motor functions that are affected by combined exposures. These data will be used to guide the assessment of children in a companion cohort study.

Ultimately, the best strategy is to develop a battery that includes both broad-based measures of cognitive and neuromotor status as well as more fine-grained assessments of specific skills. Narrow-band instruments should target domains that, based on the extant human and animal literature, are believed to be affected by exposure to the toxicant(s) in question (e.g., for hypothesis-based battery development from the perspective of childhood lead poisoning, see Dietrich et al. 2004; Rogan et al. 2001).

A second consideration in battery development is the amount of time that can be allocated to the assessment. The energy and attention spans of younger subjects are rapidly exhausted, calling for shorter periods of time for testing, more frequent breaks, and sometimes multiple appointments separated by days or weeks. Infants < 1 year of age can usually tolerate only about 45 min of standardized testing, whereas a 2-year-old can generally perform adequately over a period of roughly 75 min. For preschool and older children, breaks for a snack or just relaxing with the primary caregiver can fortify the child’s endurance. Furthermore, neurodevelopmental evaluations are often part of a larger protocol that includes collection of sociodemographic information from parents, a general medical evaluation of the child, and collection of biologic specimens such as blood, hair, and urine. Retention of children and their families in a long-term study can be compromised if the demands on their time and effort are excessive.

## Unique Considerations for Assessment of the Neonate, Infant, and Child

### The neonate.

The neonatal period extends from birth to roughly 1 month of age. Assessment of the neonate can be used to evaluate gestational maturity, neurologic status, and behavioral style and capacities. Neonatal assessment is a highly specialized area requiring continuous and careful attention to the infant’s state as the examination proceeds. Recognized infant states include several sleep states, drowsiness or transitional states, alert states, and crying. Reflexes and muscle tone, signs of stress, and alertness and orientation can all be affected by the newborn’s state.

Although neonatal neurodevelopmental assessments are not highly predictive of later functioning, performing a neurologic and neurobehavioral assessment after birth provides an assessment of the immediate effects of prenatal exposure before any influences of the extrauterine environment take place. A repeat exam (i.e., 2 or 4 weeks) allows assessment of neurobehavior after infants have stabilized. Inclusion of both assessment time points may provide insight into those effects of prenatal exposure that are transient and those that persist (Jacobson et al. 1984; Stewart et al. 2000). Neonatal assessment places special demands on quality control and site. Some instruments such as the Neonatal Behavioral Assessment Scale (NBAS) (Brazelton 1984; Brazelton and Nugent 1995) and the Neonatal Intensive Care Unit (NICU) Network Neurobehavioral Scale (Lester et al. 2004) require intensive training of examiners to master the techniques of administration and become reliable in scoring newborn responses. Training may demand travel to institutions with certification programs (for further details, see Brown Medical School Infant Development Center 2005). Each investigator will have to determine if these essential preconditions for neonatal neurobehavioral assessment can be met. The assessment of the neurologic and neurobehavioral status of the newborn will not usually be a practical option in studies involving populations that are widely dispersed geographically or living in communities lacking the required clinical facilities or trained and certified personnel.

### The infant and toddler.

Infants and toddlers between 6 and 36 months of age may be the most challenging population for obtaining systematic and reliable behavioral data using paradigms that require the child to sustain attention, follow directions, and direct their cognitive effort toward a task that requires adherence to a somewhat rigid protocol. Although the cognitive capacities of older infants and toddlers are much advanced compared with their prelinguistic and prelocomotor days, directing that cognitive capacity at any particular time is often the most challenging part of the assessment. The clinical skills of the person examining the infant and toddler are of paramount importance for obtaining data that are valid and reliable.

Indices of neurodevelopment in infants and toddlers are less stable over time and, at least before 24 months, lack substantial predictive validity for later morbidity. This is partly because of the means by which infants are able to express their cognitive abilities (i.e., primarily through sensorimotor acts) and the lack of continuity in response modalities from infancy to older childhood and adolescence. However, neurobehavioral test scores in infancy retain strong concurrent validity. Bellinger (2002) suggests that scores on infant neurodevelopmental measures can be understood in a manner analogous to the neonatologist’s interpretation of birth weight. Except in very low-birth-weight infants, weight at birth is not predictive of later weight, although birth weight is a very informative index of a newborn’s general health status. Also, as with neonatal measures, the time between neurotoxicant exposure *in utero* and postnatally and the assessment of outcome is reduced. Thus, the influence of later intervening and potentially confounding factors is diminished along with increased strength and reduced bias in the estimate of the neurotoxicant’s contributions to development (Bellinger 2002). Furthermore, where neurotoxicants such as lead, PCBs, and methylmercury have predicted poorer performance in infants and preschoolers, forward studies have demonstrated that functional deficits in neurobehavior persist into later childhood (Dietrich 2000).

### Older preschoolers and school-age children.

Because of the greater social maturity, autonomic stability, and endurance of the older preschool and school-age child, substantially more time can be devoted to a single assessment session. Also, owing to the tremendous growth in the range and clarity of a child’s response capabilities, assessments of preschoolers and older children generate a more differentiated picture of a subject’s developmental strengths and weaknesses. Functional impairments heretofore unnoticed may become apparent. To investigate the developmental effects associated with exposure to neurotoxicants in the fullness of time, studies should maintain follow-up at least until children attain school age. Indeed, recent studies of the long-term sequelae of early lead exposure would suggest follow-up to adolescence and young adulthood is necessary to reveal the full range of exposure related morbidities. There are a number of reasons for extending follow-up into late childhood and adolescence. Deficits still apparent at later ages are generally thought to be of greater practical significance because the predictive validity or prognostic value of later preschool and school-age performance is considerably greater than that of performance in infancy, thus providing a sounder basis for drawing inferences about the long-term effects of prenatal or early post-natal exposures.

## Neurodevelopmental Assessment at the Children’s Centers

Neurodevelopmental assessment practices in the longitudinal birth cohort studies at the Children’s Centers were guided by many of the principles outlined above. Most centers conducted neurodevelopmental assessments at various ages after birth (see Table 1). For the first 5 years of funding, most centers conducted assessments at 12 months and 24 months of age.

A diverse group of standardized neurodevelopmental assessment tools were employed. There were no attempts to develop common protocols among the centers conducting neuroepidemiologic studies. Differences in tests and timing of assessment reflect variations in the toxicants under study, their hypothesized effects, and practical considerations. Four centers conducted neonatal assessments; three used the NBAS (Brazelton 1984; Brazelton and Nugent 1995), and one used the NICU Network Neurobehavioral Scale (Lester et al. 2004). The Bayley Scales of Infant Development–II (Bayley 1993), which provides standard scores for mental and psychomotor development, were used by all centers at 12 months of age and by most at 24 months. All centers adjusted the child’s chronologic age for prematurity on the Bayley scales until 24 months.

A variety of other assessment tools were used to measure domains including developmental milestones, language, and behavior. A few centers employed more experimental protocols, including assessment of visual recognition memory, the autonomic nervous system, and measures of learning that parallel some of those used in animal studies. The centers at Illinois and the University of Cincinnati also assess the child’s hearing.

The choice of neurodevelopmental tests was based first on the age of the child, followed by other considerations including tests employed in previous studies, the domains of behavior thought to be affected by the main toxicant(s) under study, the availability of tests in the language of the study population, time required to administer the examination, and the test’s suitability for administration by non-doctoral-level examiners. Some centers, especially those with low-income or bilingual study populations, found it necessary to select assessment tools with a lower end of functioning range, considerably below the ages of the children being assessed.

As discussed above, it can be difficult to determine the language of assessment for children who are raised in the United States in non-English-speaking households. All centers attempted to maximize the child’s performance on all tests. One center assessed bilingual children in English. Another center based the child’s assessment on what language was usually spoken in the home. In both cases, if the examiner noted language difficulties during the exam, the tasks were readministered in the other language.

The availability of assessment tools increases with the child’s age, enhancing the researcher’s ability to focus on specific developmental domains that may be affected by the exposure of interest. Because many of the chemicals examined by these centers have not been widely studied in children (e.g., pesticides, polycyclic aromatic hydrocarbons), the choice of the domains likely to be affected may be based solely on animal studies. Finding the human analog to animal behavior is challenging. However, as previously noted, the Illinois center is addressing this problem by conducting concurrent animal studies with exposures similar to those in their human population.

All centers used some quantitative measures of the parent’s cognitive functioning and quality of caregiving in the home to control for possible confounding. In centers dealing mainly with multicultural samples, tests of adult intellectual attainment with a minimum of verbal content were used.

## Future Directions for Neurodevelopmental Assessments in the National Children’s Study

New technologies for assessment of the function and structure of the central nervous system may hold promise for advancing our understanding of the impact of environmental chemicals on neurodevelopment. Some of these are briefly discussed below.

### Computer-based experimental measures for children.

Numerous attempts have been made to develop computer-administered batteries of tests for children exposed to environmental neurotoxicants. For example, adaptations of the Neurobehavioral Evaluation System (Baker et al. 1985) have been used in studies of children exposed to heavy metals in both the United States and abroad (Dahl et al. 1996). A computer-assisted comprehensive assessment for children enrolled in environmental studies has been developed by Anger and colleagues (Rohlman et al. 2003). The Behavioral Assessment and Research System has been designed for use in studies of children as young as 3 years of age and has been adapted for use with Hispanic populations (Rohlman et al. 2001). The entire battery consists of 11 computer-administered tests assessing sustained and selective attention, working memory, motivation, new learning, response speed, executive functions, and fine-motor coordination.

The use of computer-assisted tests has several advantages in that examiner effects are reduced and data collection and scoring are automated and objective. However, care must be exercised when applying these methods to populations that have little or no exposure to computers or similar kinds of automated systems. Another potential drawback of computer-assisted tasks is that they can minimize interaction with a friendly, supportive examiner, which can be essential in helping keep the younger child motivated to complete a lengthy battery of tests. If computer-assisted tests are used, it may be best to intersperse them with examiner-administered tasks.

### Psychobiologic measures.

Studies of young children have included many psychosocial, environmental, and neurodevelopmental factors, but they rarely include individual difference measures of psychobiology. Recent studies show that children’s physiologic responses to different stressors are valid measures of individual psychobiology or autonomic reactivity (Alkon et al. 2003; Matthews et al. 1990). These psychobiology measures have been shown to affect how children interact with their family, adults, and peers at home and in school. In addition, children’s psychobiology is related to their physical and mental health (Boyce et al. 1998; Kagan et al. 1988; Porges et al. 1994). Autonomic reactivity is being studied as an outcome of organophosphate exposure prenatally and postnatally at the Berkeley Children’s Center. Autonomic dysregulation or an imbalance in the parasympathetic and sympathetic branches of the autonomic nervous system may be a sensitive indicator of acute and chronic pesticide exposure (Eskenazi et al. 1999). The Columbia center is using the orthostatic tilt test (Fifer et al. 1999) on a small subsample in the cohort. This is a noninvasive experimental paradigm used to identify infants with individual differences in autonomic regulation and diminished physiologic responses (as measured by heart rate) to blood pressure changes after postural adjustment.

### Neurobiologically based markers of development.

Several environmental studies of children have used electrophysiologic techniques to assess the effects of neurotoxicants on central nervous system function. Visual as well as auditory evoked potentials have been examined and in many cases have been found to be sensitive to environmental chemical exposures. Another promising area for the future is the use of neuroradiologic techniques such as magnetic resonance imaging (MRI). MRI assessment of brain structure and function is beginning to be used in studies of developmental neurotoxicants. In the Cincinnati center, for example, adults exposed to high levels of lead in early childhood are being examined using volumetric, functional, and spectroscopic MRI methods. These methods provide data on exposure-related structural changes, brain activation in response to standardized verbal and visual–spatial problems, and brain biochemical processes.

The use of these methods assumes the availability of a clinical facility that can provide the needed equipment and trained personnel. For many centers, particularly those far from large metropolitan areas, this will normally not be an option.

### Gene–environment interactions in neurodevelopment.

A promising area for future study is the interaction between certain genetic polymorphisms involved in neurotransmitter regulation and metabolism with environmental chemical exposures. For example, the Cincinnati center has examined the joint effects of a dopamine transporter (DAT) polymorphism and maternal prenatal smoking on childhood hyperactivity-impulsivity and inattentiveness (Kahn et al. 2003). Childhood hyperactivity–impulsivity and oppositional behaviors were associated with a DAT polymorphism, but only when the child also had in utero exposure to the products of maternal smoking. Cincinnati researchers have also identified a vitamin D receptor gene polymorphism that may be associated with greater absorption of environmental lead and thus an increased risk for neurodevelopmental toxicity (Haynes et al. 2003). The Cincinnati center is also investigating the relationship between early exposure to lead and adult criminality. The interactions of a history of early lead poisoning with four polymorphisms associated with dopamine and serotonin reception and/or transport and genes regulating monoamine oxidase A enzyme activity are being examined (Reif and Lesch 2003). Investigators at the University of California at Davis Children’s Center are investigating the interaction between specific GABA_A_ receptor polymorphisms and autism spectrum disorder (ASD). It is well known that some of the most widely used and environmentally persistent pesticides block GABA_A_ receptor chloride channels within the mammalian central nervous system, thereby producing hyperexcitability, tremors, and convulsions, depending on the level of exposure (Narahishi et al. 1998). Three genes that encode subunits of ionotropic GABA_A_ receptors cluster on chromosome 15q11–13. Maternally inherited duplications of this chromosome account for approximately 1–3% of all cases of ASD (Veenstra-VanderWeele et al. 2003). The Children’s Centers at Mount Sinai, Berkeley, and the University of Washington are examining the relationship of the paraoxynase 1 (PON1) gene in modifying the associations between organophosphate pesticide exposure and neurodevelopment. Recent evidence for a relationship between genetic background and susceptibility to the neurobehavioral toxicity of neonatal exposure to thimerosal (ethylmercury) in mice (Hornig et al. 2004) further underscores the need to consider gene–environment interactions to identify susceptible populations.

## Conclusions and Recommendations

- Long-term studies that follow participants into adolescence and early adulthood are essential to assess the full range of neurodevelopmental consequences of exposure to environmental chemicals. The vast and rapid growth of human neurobehavioral capacities from birth through early adulthood means that the functional effects of earlier damage may not be fully expressed at any given moment in time.
- Compounds targeted for investigation should be those that represent plausible neurodevelopmental hazards to fetal and postnatal central nervous system development. The compounds under investigation should represent a clear hazard to neurodevelopment on the basis of their biochemical properties. Most drugs and environmental chemicals cross the placenta with ease by virtue of their low molecular weight and many gain access to the developing central nervous system. This also requires some knowledge of the exposure levels that are likely to be encountered in the environments occupied by the samples of interest.
- Population factors including ethnicity and language must be considered in the planning of study procedures for recruitment, retention, and selection of neurodevelopmental measures. Population factors can be particularly important if substantial confounding and multicultural issues are involved. These features become crucial when it comes to the choice of appropriate neurodevelopmental measures that will accurately reflect the cognitive and sensorimotor capacities of the population under investigation. Ideally, examiners familiar with the language and culture of the population under study should be employed.
- The proposed work should be compatible with the physical and human resources at hand. Sites faced with populations that are widely dispersed geographically, having limited transportation, or lack of trained personnel will be restricted in the depth of neurodevelopmental assessments that can be realistically implemented.
- Neuropsychologic batteries employed in these studies should include a balance of those with measures both broad and narrow in scope. Batteries too narrow in scope can easily miss deficits associated with environmental chemical exposures. On the other hand, fine-grained measures of more narrowly defined neurobehavioral domains can shed light on exposure-specific effects and brain–behavior relationships. If specific neurobehavioral domains are targeted for assessment, their selection should be guided by hypothesis-driven inference. An assessment battery that includes both broad- and narrow-band instruments will usually be optimal.
- Procedures for monitoring the quality of data collection and scoring should be maintained throughout the life of the study. Monitoring of interexaminer reliability and proficiency should be practiced on a regular basis. In the event that multiple examiners are required at a single center, their training and examination techniques should be explicitly checked, monitored, and quantitatively recorded. In multicenter studies, training should be standardized across sites. This may require annual meetings and more frequent conference calls to discuss issues related to administrations and scoring of tests.
- Data safety and monitoring procedures must be in place. As health care professionals as well as researchers, we have a duty to treat study participants with respect and concern. Referral of infants and children presenting with suspect neurologic or developmental signs or symptoms should be made through the primary care provider. In every case, consultation with the primary caregiver is mandatory.
- The unique needs of study participants involved in neurodevelopmental assessment from birth through adulthood must be considered. Every age presents special challenges. Assessment of the neonate calls for extensive training of the examiner(s) and a highly controlled environment. Infants and toddlers present special challenges with regard to directing their attention to the demands of cognitive and sensorimotor tasks. Assessment of school-age children and adolescents provides an opportunity to examine a more differentiated picture of participants’ developmental strengths and weaknesses. Assessments of the older adolescent and young adult call for an approach that recognizes a participant’s legal rights as a research subject and sometimes requires special measures to assure subject protection and confidentiality of any data that may be obtained.
- New approaches and technologies should be exploited in future studies. Computer-based assessment, psychobiologic measures, neural markers of central nervous system activity, and especially the promise of advances in human genetics have the potential to lead to substantial progress in our understanding of the effects of environmental chemicals on fetal and child development.

### Final remarks.

A battery of tests that can be universally regarded as valid and reliable for evaluating the potential impact of neurotoxicants on the developing central nervous system does not exist. Specific approaches and tests have been reviewed and recommended based on the authors’ experiences and ongoing work in the Children’s Centers and other studies. The quandary faced by both seasoned investigators and researchers new to the area when planning a study was summarized by one experienced observer: “I cannot recommend any tests in this endeavor; many are appropriate. Overall strategy, a principled theoretical framework, and adequately specified domains are what count, not tests” (Bernstein 1994).

The vulnerability of the developing human central nervous system to environmental chemical compounds has been well established. The contribution of these exposures in utero or postnatally to the development of disorders of uncertain etiology such as attentional deficit hyperactivity disorder or pervasive developmental disorder/autism is not known at present. Large-scale human studies such as the National Children’s Study may provide some clues. In the final analysis, the human neurodevelopmental phenotype will be more fully revealed in large-scale studies that take account of environmental chemical influences, the social milieu, and complex human genetic characteristics that we are just beginning to understand (Hamer 2002).

## Figures and Tables

**Table 1:** Neurodevelopmental assessment in the Children’s Centers for the first 5-year funding cycle.

Assessment tool	University of Illinois	University of California, Berkeley	Columbia University	Mount Sinai Medical Center	University of Cincinnati
Neonatal assessment tools					
Brazelton (Brazelton and Nugent 1995)	Birth	Birth		Birth	
Ballard (Ballard et al. 1991)				Birth	
NICU Network Neurobehavioral Scale (Lester et al. 2004)					Birth, 4 weeks
Infant and toddler assessment tools					
Bayley scales (Bayley 1993)	6, 12 months	6, 12, 24 months	12, 24, 36 months	12, 24^a^ months	12, 24 months
PLS (Zimmerman et al. 1992)		6, 12, 24 months			
CBCL (Achenbach and Rescorla 2004)		12, 24 months			
Denver (Frankenburg et al. 1992)			6 months		
Fagan (Fagan et al. 1986)	6 months		6 months		
Infant Behavior Questionnaire (Rothbart 1981)				12 months	
Toddler Behavior Assessment Questionnaire (Goldsmith 1996)				24^a^ months	
Sleep questionnaire (adapted from Morrell 1999)					6, 12, 18, 23 months
Cognitive tasks^b^ (Aguiar and Baillargeon 1999)	6, 9, 12 months				
Child assessment tools					
WPPSI-III (Wechsler 2002)		42^c^ months	60 months		
NEPSY (Korkman et al. 1998)		42^c^ months			
McCarthy (McCarthy 1974)		42^c^ months			
CBCL (Achenbach and Rescorla 2004)		42^c^ months			
Other child assessments					
Auditory assessment	Birth, 12 months				
Autonomic Nervous System Assessment (Alkon et al. 2003)		6, 12, 42^c^ months			
HOME Scale (Caldwell and Bradley 1984)		6, 12, 24, 42^c^ months	36 months	12, 24 months	12 months
Maternal intelligence					
PPVT (Dunn and Dunn 1981)/TVIP (Dunn et al. 1986)		6 months		Pregnancy	
WASI (Wechsler 1999)					12 months
TONI-2 (Brown et al. 1990)			24 or 36 months		
WAIS-III (Wechsler 1997)	Enrollment (matrices and block design)	6 months (matrices)			

Abbreviations: CBCL, Child Behavior Checklist; HOME, Home Observation for Measurement of the Environment; NEPSY, A Developmental Neuropsychological Assessment; PLS, Preschool Language Scale; PPVT, Peabody Picture Vocabulary Test; TONI-2, Test of Nonverbal Intelligence-2; TVIP, Test de Vocabulario en Imagenes Peabody; WAIS-III, Wechsler Adult Intelligence Scale-III; WASI, Wechsler Abbreviated Scales of Intelligence; WPPSI-III, Wechsler Preschool and Primary Scales of Intelligence-III.

a Children were brought in up to 5 years of age for the “24-month” assessment; if too old for the Bayley, they were assessed with the Batelle Developmental Inventory (Newborg et al. 1984).

b This consists of object retrieval tasks that examine working memory, executive control, and cognitive development.

c Currently underway as part of second 5-year funding cycle.
